# The association between physical activity and delayed neurocognitive recovery in elderly patients: a mediation analysis of pro-inflammatory cytokines

**DOI:** 10.1007/s40520-024-02846-z

**Published:** 2024-09-11

**Authors:** Junfang Niu, Yanan Li, Qi Zhou, Xiang Liu, Peixia Yu, Fang Gao, Xia Gao, Qiujun Wang

**Affiliations:** 1 Department of Anesthesiology, Hebei Medical University Third Hospital, Shijiazhuang, China; 2 Department of Epidemiology and Statistics & Hebei Province Key Laboratory of Environment and Human Health, School of Public Health, Hebei Medical University, Shijiazhuang, China

**Keywords:** Delayed neurocognitive recovery, Physical activity, Sedentary behavior, Elderly patient, Pro-inflammatory cytokines

## Abstract

**Background:**

Delayed neurocognitive recovery (dNCR) can result in unfavorable outcomes in elderly surgical patients. Physical activity (PA) has been shown to improve cognitive function, potentially by reducing systemic inflammatory responses. However, there is a lack of supportive data indicating whether PA has a protective effect against dNCR.

**Aims:**

To examine the correlation between dNCR and PA, and to further analyze if pro-inflammatory cytokines mediate this relationship.

**Methods:**

This study is a prospective nested case-control investigation of elderly patients who had knee replacement surgery. dNCR was defined as a decline in cognitive function compared with baseline by using a battery of neuropsychological tests. PA was assessed with the Physical Activity Scale for the Elderly (PASE). Enzyme-linked immunosorbent assay (ELISA) was used to measure the serum concentrations of IL-6, IL-1β, and TNF-α. Multivariable logistic regression analysis was conducted to assess the association between PA and dNCR. Mediation analysis was employed to evaluate whether pro-inflammatory cytokines mediate the relationship between them.

**Results:**

A cohort of 152 patients was included, resulting in an incidence rate of dNCR of 23.68%. PA was associated with dNCR after full adjustment [OR = 0.199, (95% CI, 0.061; 0.649), *P* = 0.007]. Mediation analysis showed that the IL-6 mediated the statistical association between PA and dNCR, with mediation proportions (%) of 77.68 (postoperative concentration of IL-6) or 27.58 (the absolute change in IL-6 before and after surgery).

**Conclusions:**

PA serves as a protective factor against dNCR, possibly through the reduction of pro-inflammatory cytokine concentrations.

**The Chinese Clinical Trail Registry:**

: www.http://chictr.org.cn, Registration No. ChiCTR2300070834, Registration date: April 24, 2023.

**Supplementary Information:**

The online version contains supplementary material available at 10.1007/s40520-024-02846-z.

## Introduction

As life expectancy increases, the world’s elderly population is growing [1] and more than 60 million patients worldwide undergo surgical treatment yearly [2]. Perioperative neurocognitive disorders are common complications, which serve as overarching terms for cognitive impairments identified in the preoperative or postoperative period. Cognitive decline diagnosed within 30 days after surgery is called delayed neurocognitive recovery (dNCR) [3]. The main manifestations are impaired memory, comprehension, and attention in patients after surgery and anesthesia. The incidence of dNCR ranges from 25.8% [4] to 41.4% [5] at 1 week after major noncardiac surgery or at discharge. This severely affects the patient’s quality of life, prolongs their hospital stay, increases medical costs, and raises the long-term risk of cognitive disorders such as dementia [6]. However, the current understanding of the mechanisms is still unclear, and there are no specific treatments. Thus, early preventive measures and enhancing cognitive reserve are crucial.

The neuroinflammatory response is one of the mechanisms being investigated [7, 8]. Peripheral inflammation caused by trauma or surgical insults triggers an increase in the expression of inflammatory cytokines by macrophages, particularly IL-6, IL-1β, and TNF-α [9, 10]. Inflammatory cytokines can enter the central nervous system via the vagus nerve, through a compromised blood-brain barrier, or directly into the peri arachnoid spaces of the brain, where they can stimulate microglia to produce pro-inflammatory cytokines, leading to neurotoxic symptoms and cognitive dysfunction [11, 12]. Animal experiments have detected increased levels of inflammatory cytokines (IL-1β, IL-6, and TNF-α) in the central nervous system following non-craniotomy surgery, positively correlated with the levels in peripheral blood [13]. This finding further corroborates the mechanism by which peripheral injury incites a central inflammatory response. Regulation of these inflammatory cytokines may improve postoperative neurocognitive function.

Physical activity (PA) is an acknowledged strategy for reducing the risk of age-related diseases [14]. Insufficient PA is associated with an increased risk of diseases such as Alzheimer’s and dementia [15, 16]. Higher levels of habitual PA are associated with a 14–24% reduction in dementia risk and a 33–48% reduction in cognitive risk [17]. Some studies have shown that exercise can improve the inflammatory state of the body and reduce levels of inflammatory biomarkers such as CRP, IL-1β, TNF-α, and IL-6 [18–20].

However, there is little research on whether PA offers a protective effect against dNCR. Elderly patients often have limited PA, particularly those with chronic conditions such as osteoarthritis in the lower limbs. The authors examined the primary hypothesis that light to moderate PA was associated with dNCR. The secondary hypothesis was that pro-inflammatory cytokines might mediate this association.

## Materials and methods

### Institutional review board and consent

The study was approved by the Third Hospital of Hebei Medical University (No. 2023-018-1, approval date: March 21, 2023) and was registered on the Chinese Clinical Trial Registry (http://www.chictr.org.cn, registration No. ChiCTR2300070834, registration date: April 24, 2023). Written informed consent was obtained from all participants. This manuscript adheres to the recommendations of the Strengthening the Reporting of Observational Studies in Epidemiology (STROBE) initiative.

### Study design and participants

This study employed a prospective nested case-control design. Between April 25 and August 30, 2023, a total of 694 patients at the Third Hospital of Hebei Medical University underwent unilateral knee arthroplasty. The inclusion criteria were as follows: (1) patients aged 60 to 90 years under a combination of general anesthesia and nerve block; (2) American Society of Anesthesiologists (ASA) classification of I to III; (3) body mass index (BMI) between 18 and 30 kg/m²; and (4) willingness to participate in the study and the ability to communicate effectively. The exclusion criteria were: (1) a Mini-Mental State Examination (MMSE) score below 24; (2) preexisting central nervous system disorders, including cerebrovascular and neurodegenerative diseases; (3) continuous use of sedatives, antipsychotics, anti-inflammatory drugs, or anticholinergic medications; (4) patients with severe hearing or vision impairments; and (5) preoperative systolic blood pressure exceeding 190 mmHg or diastolic blood pressure exceeding 100 mmHg. The criteria for elimination included: (1) patients who temporarily canceled their surgery or changed the anesthesia method; (2) those with incomplete or inadequate blood sample collection; and (3) patients who developed postoperative severe systemic inflammatory infections. One researcher assessed the eligibility of each patient, collected relevant clinical data, and conducted assessments of PA. Another researcher, blinded to the patient’s PA and other clinical information, was responsible for conducting the neuropsychological assessment.

### Perioperative care

All patients were managed following the institution’s standardized protocols. Before the surgical procedure, patients were instructed to refrain from food and drink for eight hours. Thirty minutes before the surgical procedure, an ultrasound-guided femoral nerve block was administered using 20 ml of 0.375% ropivacaine. Anesthesia induction was facilitated with midazolam (0.05–0.07 mg/kg), sufentanil (0.2–0.4 µg/kg), propofol (2-2.5 mg/kg), and rocuronium (0.6 mg/kg). Anesthesia was maintained with propofol and remifentanil, with dosages adjusted based on vital signs and surgical stimulation, to maintain a bispectral index (BIS) of 40–60. Invasive arterial blood pressure monitoring was conducted through the radial artery to maintain blood pressure within 20% of baseline levels. There were no limitations on the administration of vasoactive medications. Within the first 48 h postoperatively, all patients received patient-controlled intravenous analgesia (PCIA) with the same types and dosages of medication. And the postoperative Visual Analogue Scale (VAS) scores were also assessed and recorded.

### Exposures: assessment of PA

The Physical Activity Scale for the Elderly (PASE) is a widely utilized instrument in epidemiological research for the assessment of PA in older adults. Its reliability and validity have been consistently confirmed by various studies [21, 22]. This scale integrates data on leisure, household, and work-related activities, and can be completed in a relatively brief period, typically around five minutes. Leisure activities encompass a range of activities, including outdoor walking, exercises of varying intensities, recreational activities, and muscle strengthening, among others. The frequency of participation is recorded as never, seldom (1–2 days per week), occasionally (3–4 days per week), or often (5–7 days per week). The duration of each activity is quantified by the mean time spent on it daily. Only those activities associated with PA at work are recorded, with the time spent on these activities calculated about the number of weekly working hours. Household labor, including light and heavy housework, gardening, home maintenance, and caregiving, is recorded in a binary format of yes/no, with no specific guidelines for the frequency and duration of household activities. The cumulative PASE score is calculated based on the weights of activity items established by the scale’s creators [22].

In the present study, two forms of categorization were employed to distinguish patients’ physical activity levels: a continuous variable and a binary variable. The continuous variable represents the total weighted activity score. By contrast, the binary variable employs a second-order clustering method to categorize patients into physical activity and sedentary behavior (Supplementary Fig. 1).

### Outcomes: neuropsychological test battery and diagnosis of dNCR

Neuropsychological evaluations were conducted one to three days before surgery and five to seven days after or after discharge. Before assessment, the Confusion Assessment Method (CAM) scale was employed to exclude patients who were in a delirious state. To minimize the impact of learning effects and reduce the overall testing time, a dual-version test format was employed for the assessment scale. The test sequence included immediate word recall at the outset and delayed word recall at the conclusion, with the remaining tests following a fully Latin square design. The assessments were conducted in a quiet room, with only the evaluating physician and the patient present.

The assessment methodology utilized a neuropsychological test battery similar to that of the International Study of Postoperative Cognitive Dysfunction (ISPOCD) research group [23, 24], comprising: (1) Rey Auditory Verbal Learning Test (RAVLT) [25]: Comprising word learning and word recall sections, the test recorded the number of words correctly recalled immediately and after a 20-minute delay; (2) Trail Making Test (TMT) [26]: Consisting of Parts A and B, the test recorded the time taken to complete each part; (3) The Symbol Digit Substitution Test (SDST) [27]: recorded the number of correct substitutions made by the patient within 90 s; (4) Part C of the Stroop Color-Word Interference Test (SCWT) [28]: recorded the time taken made by the patient when reading 20 color-word interferences.

To minimize the potential for redundancy within the cognitive tests and reduce the data’s dimensionality, the six scores obtained from the tests were subjected to principal component analysis with orthogonal rotation (a linear transformation of the data) [29]. This resulted in the identification of four uncorrelated factors, which collectively represent four primary cognitive domains: (1) Verbal Learning; (2) Verbal Memory; (3) Executive Function; and (4) Attention/Concentration. The utilization of the baseline rotation factor scores of the subjects to determine the scoring coefficients at all time points ensures consistency within the cognitive domain over time. The dNCR is defined as a decrement of at least one standard deviation or more in at least one of the four cognitive domains, irrespective of the presence or absence of concomitant functional impairments in daily living activities [3].

### Mediator variable: detection of pro-inflammatory cytokines

The plasma samples were collected to detect the levels of IL-6, IL-1β, and TNF-α before surgery and one day after the surgery. The blood samples were incubated at room temperature for 30 min. Subsequently, centrifugation at 3,000 g for 15 min at 4 °C was performed to separate the serum, which was then stored at -80 °C. The levels of IL-6 (EK106, Multi-Sciences, China), IL-1β (EK101BHS, Multi-Sciences, China), and TNF-α (EK182, Multi-Sciences, China) were quantified using ELISA kits following the manufacturer’s instructions.

### Covariates

Potential confounders and effect modifiers were identified from guidelines or expert consensus documents [30, 31] related to postoperative cognitive function. Patient demographics, medical history, lifestyle, and laboratory test results were documented from the clinical case management system or self-reported by the patients preoperatively. Specific indicators were shown in Table 1.

**Table 1:** Baseline characteristics, laboratory examination and the distribution of dNCR

Variables	No- dNCR (*n* = 116)	dNCR (*n* = 36)	t/Z/χ2	*P*
Age, year	66.5(6)	71(5)	-4.322	**< 0.001**
Gender, n(%)			0.026	0.871
Female	92(79.30)	29(80.60)		
Male	24(20.70)	7(19.40)		
Body mass index, kg/m2	26.83(3.91)	26.69(4.91)	-0.171	0.864
Education, n(%)			1.332	0.248
Less than high school	75(64.70)	27(75.00)		
At least high school	41(35.30)	9(25.00)		
Two or more surgeries in the past year, n(%)			-	0.291
No	109(94.00)	32(88.90)		
Yes	7(6.00)	4(11.10)		
Smoking, n(%)			-	0.164
No	97(83.60)	34(94.40)		
Yes	19(16.40)	2(5.60)		
Drinking, n(%)			-	0.122
No	102(87.90)	35(97.20)		
Yes	14(12.10)	1(2.80)		
CCI, n(%)				**< 0.001**
0	52(44.80)	4(11.10)		
1	45(38.80)	18(50.00)		
2	14(12.10)	6(16.70)		
3	5(4.30)	4(11.10)		
4	0(0.00)	4(11.10)		
ASA, n(%)			8.654	**0.003**
II	111(95.70)	29(80.60)		
III	5(4.30)	7(19.40)		
CES-D 10 score, point	7(2)	9(1)	-5.419	**< 0.001**
PSQI score, point	8(3)	8(2)	-0.040	0.968
MMSE score, point	27.5(3)	26(2.75)	-2.752	**0.006**
PASE score, point	149.75(131.63)	70.25(66.13)	-4.404	**< 0.001**
PASE categorization, point				**< 0.001**
Sedentary behavior	42(36.20)	29(80.60)		
Physical activity	74(63.80)	7(19.40)		
Duration of anesthesia, min	150(30)	150(30)	-0.406	0.685
Duration of surgery, min	90(36)	89.5(29.75)	-0.135	0.893
Estimated blood loss, ml	155(100)	150(100)	-0.391	0.696
VAS, point	2(1)	2(1)	-0.795	0.427
ADL score, point	100(10)	95(10)	-2.682	**0.007**
Length of hospital stay, day	8(3)	8(4)	-1.317	0.188
**Laboratory examination**				
preop IL-1β, pg/ml	3.53(1.43)	4.24(1.65)	-2.168	**0.030**
postop IL-1β, pg/ml	7.76 ± 2.38	8.20 ± 2.53	-0.944	0.346
ΔIL-1β, pg/ml	4.12 ± 2.40	4.09 ± 2.52	0.050	0.960
%Chg IL-1β	1.17(1.15)	0.99(1.38)	-0.758	0.448
preop IL-6, pg/ml	9.83(7.46)	15.02(11.10)	-2.590	**0.010**
postop IL-6, pg/ml	19.87(12.06)	32.81(9.88)	-5.422	**< 0.001**
ΔIL-6, pg/ml	8.71(12.04)	18.44(6.92)	-4.676	**< 0.001**
%Chg IL-6	0.81(1.37)	1.29(2.08)	-2.646	**0.008**
preop TNF-α, pg/ml	21.69(6.46)	25.90(13.33)	-3.767	**< 0.001**
postop TNF-α, pg/ml	32.85(8.64)	35.85(11.98)	-3.262	**0.001**
ΔTNF-α, pg/ml	8.33 ± 7.29	8.84 ± 8.80	-0.345	0.731
%Chg TNF-α	0.40 ± 0.35	0.36 ± 0.35	0.548	0.585

**Abbreviations** dNCR, delayed neurocognitive recovery; CCI: Charlson Comorbidity Index; ASA: American Society of Anesthesiologists; CES-D 10: the 10-item Center for Epidemiologic Studies Depression Scale; MMSE, Mini Mental State Examination; PASE: physical activity scale for the elderly; PSQI: Pittsburgh Sleep Quality Index; MMSE: Mini-Mental State Examination; VAS: visual analogue scale; ADL: Activities of Daily Living; preop: preoperative; postop: postoperative; Δ : absolute change (postoperative - baseline); %Chg: relative changes [(postoperative - baseline)/baseline]

**Notes** Mean ± SD or Median (interquartile range) for continuous variables; *n*(%) for categorical variables; The *P* values were calculated by *t*-test, Mann-Whitney *U* test, chi-squared test or Fisher’s exact test. Boldface values indicate *P* < 0.05

**Table 2:** Logistic regression model showing associations between PA and dNCR

	Model 1		Model 2		Model 3	
	Crude OR (95% CI)	*P*	Adjusted OR (95% CI)	*P*	Adjusted OR (95% CI)	*P*
Age	1.215 (1.106, 1.336)	**< 0.001**	1.216 (1.054, 1.403)	**0.007**	1.226 (1.068, 1.407)	**0.004**
CCI	2.325 (1.553, 3.483)	**< 0.001**	2.407 (1.384, 4.186)	**0.002**	2.292 (1.322, 3.973)	**0.003**
CES-D 10	2.171 (1.550, 3.041)	**< 0.001**	2.632 (1.625, 4.264)	**< 0.001**	2.354 (1.530, 3.623)	**< 0.001**
ASA	5.359 (1.585, 18.119)	**0.007**	4.952 (0.870, 28.189)	0.071	4.395 (0.883, 21.881)	0.071
MMSE	0.853 (0.724, 1.006)	0.059	-	-	-	-
ADL	0.985 (0.949, 1.022)	0.410	-	-	-	-
PASE Score	0.986 (0.980, 0.993)	**< 0.001**	0.986 (0.976, 0.995)	**0.002**	-	-
Physical activity vs. Sedentary behavior	0.137 (0.055, 0.340)	**< 0.001**	-	-	0.199 (0.061, 0.649)	**0.007**

**Abbreviations** PA: physical activity; dNCR, delayed neurocognitive recovery; CCI: Charlson Comorbidity Index; CES-D 10: the 10-item Center for Epidemiologic Studies Depression Scale; ASA: American Society of Anesthesiologists; MMSE: Mini-Mental State Examination; ADL: Activities of Daily Living; PASE: physical activity scale for the elderly; CI: confidence interval; OR: odds ratio

Model 1: Univariate logistic regression analysis. Boldface values indicate *P* < 0.05

Model 2: Multivariate logistic regression analysis was conducted with the PASE score as a continuous variable, adjusting for age, CCI, CES-D 10, and ASA

Model 3: Multivariate logistic regression analysis was performed after categorizing the PASE scores into two clusters, ‘Physical Activity’ and ‘Sedentary Behavior’, adjusting for age, CCI, CES-D 10, and ASA

### DAGs

**Fig. 1:**
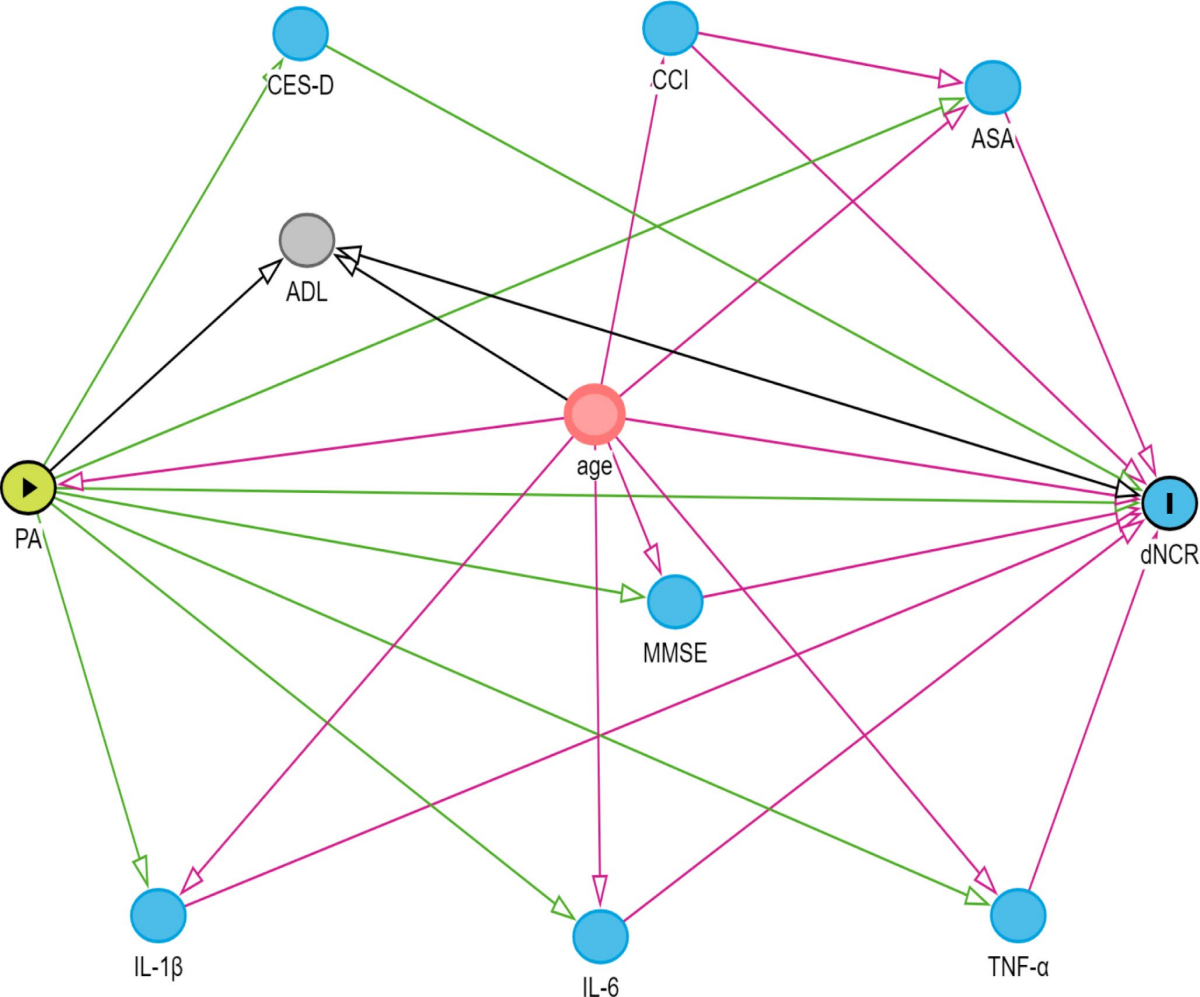
Directed Acyclic Graph (DAG) Green circles represent exposures, blue circles represent outcomes, red circles represent common ancestors of exposure and outcomes (i.e. confounding factors), light blue circles represent ancestors of the outcome (i.e. causal determinants of the outcome), and brown circles represent other variables. Green lines depict causal pathways from exposure, while red lines indicate biased pathways to the outcome. Variables preceding arrows pointing to exposures or outcomes signify potential influencing factors, and variables linking exposures to outcomes may serve as mediating variables

Using the online Directed Acyclic Graphs (DAGs) tool DAGitty (https://www.dagitty.net), we can establish the causal logical relationships between relevant variables to select independent variables and control for confounding factors [32] (Fig. 1).

### Sample size calculation

The calculation of the sample size was conducted using PASS software (version 11.0.7; NCSS, LLC., United States) to ascertain the anticipated effect size, with a significance level set at 0.05 and the statistical power at 90%. In alignment with the findings from the ISPOCD study, the incidence of dNCR within one week following non-cardiac surgery in subjects aged 60 years and older was noted to be 25.8% [4]. The anticipated odds ratio (OR), indicative of the influence of PA on dNCR, was adopted from prior literature, where an OR of 0.26 was reported, with the control group having an 80% exposure to PA [33]. At least 140 cases of patients are required for statistical requirements.

### Statistical analysis

The analytical process aimed to address three interrelated questions: (1) the correlation between PA and dNCR; (2) the correlation between pro-inflammatory cytokines and dNCR after controlling for PA and other risk factors; (3) the mediation of the relationship between PA and dNCR by pro-inflammatory cytokines.

The IBM SPSS Statistics software (version 26.0.0.0; IBM Corporation, Armonk, United States) was used for statistical analyses. Data were presented as mean ± standard deviation for normally distributed continuous variables and median (interquartile range) for non-normal distributions. Categorical variables were reported as frequencies (%). Serum IL-6, IL-1β, and TNF-α levels were analyzed in four forms: preoperative, postoperative, absolute change (postoperative minus preoperative), and relative change [(postoperative minus preoperative)/preoperative]. Comparative analyses between groups were conducted using *t*-tests, Mann-Whitney U tests, chi-square tests, and Fisher’s exact tests, as warranted.

Univariate logistic regression was initially performed to screen for prognostic indicators associated with dNCR. Variables with a *p*-value below 0.05 were considered for a multivariate regression model. The variance inflation factor was used to assess multicollinearity between variables. The model’s fit was evaluated through the Hosmer-Lemeshow test, and its discriminatory ability was determined by the area under the receiver operating characteristic (ROC) curve. All *p*-values were calculated using a two-tailed approach, with a significance level of 0.05. We conducted a sensitivity analysis by categorizing the PASE-weighted total score through median and tertiles. Additionally, patients under the age of 65 were excluded to observe the correlation between PA and dNCR.

The mediation analysis was conducted using the SPSS PROCESS macro developed by Andrew F. Hayes (https://processmacro.org/index.html), utilizing Model 4 to explore the mediation pattern. Mediation effect, direct effect, and proportion mediated were calculated. This strategy used statistical evidence to elucidate potential mechanisms. In this case, the direct effect indicated the association between PA and dNCR, while the mediation effect elucidated the mediation of pro-inflammatory cytokines in this association. The proportion mediated described the percentage of the mediation effect. Parameter estimates were performed using bias-corrected non-parametric percentile bootstrapping. If the 95% confidence interval (CI) did not include zero, the coefficients and indirect effects were considered significant.

## Results

### Patient characteristics

In a cohort of 694 patients who underwent unilateral knee arthroplasty, 175 surgical patients were identified and included in the study based on the established criteria. However, 23 participants were subsequently eliminated for various reasons: 4 patients had their surgeries suspended, 10 had inadequate or improperly collected blood samples, 6 did not complete postoperative neuropsychological assessments, and 3 developed systemic infection symptoms postoperatively. Ultimately, 152 patients were included in the study, providing complete medical history follow-up information and blood specimen test data. Among them, 36 patients experienced dNCR in the early postoperative period, leading to an incidence rate of 23.68%.

The baseline characteristics of the patients are presented in Table 1. The median age of the participants was 71 years old, with an interquartile range of 63 to 73 years. The gender distribution was predominantly female, accounting for 80.26% of the participants. Despite this notable gender imbalance, the current data analysis reveals no significant difference in the incidence of dNCR between the genders. Baseline data revealed significant differences between the two groups regarding age, CCI, ASA, CES-D 10, MMSE, and physical activity (*P* < 0.05). Regarding pro-inflammatory cytokines, significant differences were observed between the two groups in terms of preoperative IL-1β, preoperative and postoperative IL-6, as well as both absolute and relative variations in IL-6, preoperative and postoperative TNF-α (*P* < 0.05).

### PA is Associated with dNCR

We found a significant association between PA and dNCR in the early postoperative period [PA for continuous variable: OR = 0.986, (95%CI: 0.980; 0.993), *P* < 0.05] (Model 1). After adjusting for age, CCI, ASA, and CES-D 10, the trend remained consistent [OR = 0.986, (95% CI: 0.976; 0.995), *P* < 0.05] (Model 2). After categorizing the PASE scores through second-order cluster analysis, the incidence rate of dNCR was significantly reduced in the physical activity group compared to the sedentary behavior group [OR = 0.137, (95% CI: 0.055; 0.340), *P* < 0.05] (Model 1). Upon further adjustment for the same factors, the trend remained consistent [OR = 0.199, (95% CI: 0.061; 0.649), *P* < 0.05] (Model 3). The positive predictive ability of the multivariate logistic regression model 2 and 3 validated by ROC analysis is indicated by the area under the curve of 0.93 (95% CI: 0.89; 0.97) and 0.92 (95% CI: 0.88; 0.96), respectively (Supplementary Fig. 2). To further validate the robustness of the results, we conducted three sensitivity analyses (Supplementary Table 1), which indicated that the association between PA and dNCR remained significant.

### Pro-inflammatory cytokines were associated with dNCR

In the univariate logistic regression analysis, pro-inflammatory cytokines that achieved statistical significance (*P* < 0.05) were subjected to multivariate logistic regression analysis. Models 7–13 in Table 3 sequentially represent the multivariate regression analysis of the three pro-inflammatory factors after full adjustment. When PA was considered as a continuous variable, postoperative IL-6 levels, the absolute and relative changes in IL-6 retained statistical significance (*P* < 0.05). When PA was treated as a binary variable, postoperative IL-6 levels, the absolute change in IL-6, and preoperative TNF-α were statistically significant (*P* < 0.05).

**Table 3:** Multivariate logistic regression model showing associations between pro-inflammatory cytokines and dNCR

		PASE score^#^		Physical Activity vs. Sedentary Behavior^*^	
		Adjusted OR (95% CI)	*P*	Adjusted OR (95% CI)	*P*
Model 7	Preop IL-1β	0.969 (0.574, 1.636)	0.906	0.964 (0.564, 1.646)	0.893
Model 8	Preop IL-6	0.966 (0.841, 1.110)	0.629	1.032 (0.918, 1.161)	0.599
Model 9	Postop IL-6	1.107 (1.023, 1.197)	**0.011**	1.120 (1.035, 1.213)	**0.005**
Model 10	Δ IL-6	1.091 (1.017, 1.171)	**0.015**	1.085 (1.013, 1.163)	**0.020**
Model 11	%Chg IL-6	1.792 (1.061, 3.028)	**0.029**	1.494 (0.958, 2.328)	0.076
Model 12	Preop TNF-α	1.090 (0.993, 1.197)	0.071	1.098 (1.003, 1.203)	**0.043**
Model 13	Postop TNF-α	1.065 (0.967, 1.173)	0.200	1.079 (0.979, 1.189)	0.127

**Abbreviations** dNCR, delayed neurocognitive recovery; PASE: physical activity scale for the elderly; preop: preoperative; postop: postoperative; Δ: absolute change (postoperative - baseline); %Chg: relative changes [(postoperative - baseline)/baseline]; CI: confidence interval; OR: odds ratio

^#^ PA was treated as a continuous variable

^*^ PA was categorized as a binary variable

**Models 7–13** Adjusting for PA, age, ASA, CCI, and CES-D 10. Boldface values indicate *P* < 0.05

### Pro-inflammatory cytokines mediate the relationship between PA and dNCR

**Fig. 2:**
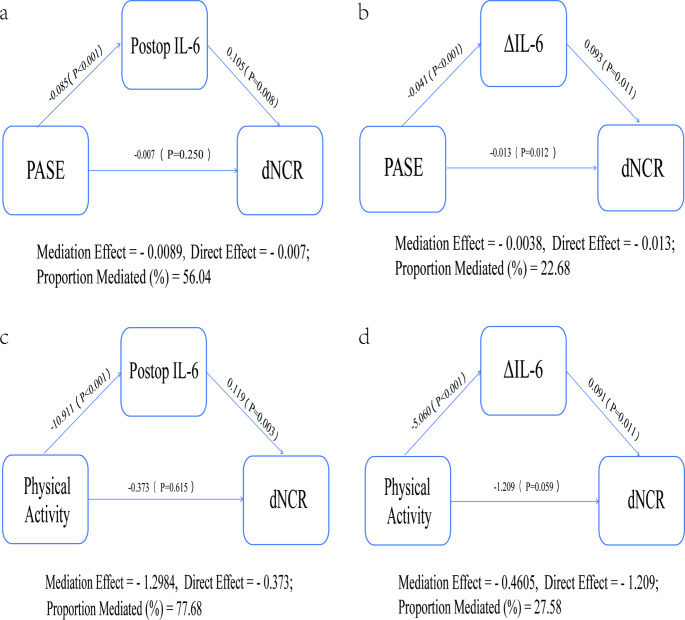
Mediation models of PA, pro-inflammatory biomarkers, and dNCR after adjusting for age, ASA, CCI, and CES-D 10 The graphs in a and b represent the mediating roles of postoperative IL-6 and the absolute change in IL-6, respectively, with PASE as a continuous variable. In contrast, graphs c and d represent the mediation models for patients with active labor compared to those with a sedentary lifestyle, when PASE is considered as a binary variable. Direct effect refers to the relationship between PA (exposure) and dNCR (outcome). The mediation effect refers to the relationship between PA (exposure) and IL-6 (mediator), as well as the relationship from IL-6 (mediator) to dNCR (outcome). Proportion mediated (%) = Mediation effect / (Direct effect + Mediation effect)

These findings led us to explore whether pro-inflammatory cytokines mediated the relationship between PA and dNCR. Notably, through mediation analysis, we observed that both postoperative IL-6 and the absolute change in IL-6 significantly mediated this relationship (Fig. 2). When PA was treated as a continuous variable, after adjusting for age, ASA, CCI, and CES-D 10, postoperative IL-6 and the absolute change in IL-6 accounted for 56.04% and 22.68% of the association, respectively. As PA was considered as a binary variable, with the same adjustments, postoperative IL-6 and the absolute change in IL-6 explained 77.68% and 27.58% of the association, respectively. The mediating effect of postoperative IL-6 was particularly pronounced.

## Discussion

Our research investigated the relationship between PA and dNCR in elderly patients undergoing total knee arthroplasty. The study revealed a significant correlation between the two variables, with the association being significantly mediated by serum pro-inflammatory cytokines, particularly IL-6. The findings suggest that engaging in preoperative PA may confer a protective effect against dNCR, potentially by reducing the levels of serum pro-inflammatory factors.

In this study, we observed an incidence of dNCR of 23.68%. This rate is comparable to the 25.8% incidence of cognitive dysfunction reported within one week after non-cardiac surgery in the ISPOCD1 multicenter study [4]. However, Monk et al. [5], employing a similar study design, noted a higher incidence rate of cognitive dysfunction at 41.4%. The lower incidence rate observed in our study could be due to the strict control of potential confounding factors at the outset of the study and slight differences in the definition of dNCR. Additionally, among the 175 patients ultimately included in the study, 23 cases were excluded for various reasons. Of these, 14 cases were randomly missing data (including 4 patients whose surgeries were postponed and 10 with inadequate or improperly collected blood samples). Another 9 cases had potentially non-random missing data, which constituted approximately 5% of the total sample size. Consequently, no special statistical treatment was applied to the missing data.

Neuroinflammatory response is widely recognized as a major mechanism of postoperative cognitive dysfunction [12, 34]. Our previous research findings [35], as well as the results of this study, have both confirmed this perspective. Elevated levels of pro-inflammatory cytokines in the circulation, such as TNF-α, can disrupt the blood-brain barrier and promote the migration of monocytes/macrophages to the brain parenchyma [36]. Animal experiments have also indicated that an increase in pro-inflammatory cytokines, such as IL-6, can decrease the expression of tight junction proteins in the blood-brain barrier [37], leading to its impairment. Pro-inflammatory cytokines can cross the impaired blood-brain barrier and activate central astrocytes and microglia. This increases pro-inflammatory cytokines, such as IL-1β and TNF-α, causing neuroinflammation and neuronal apoptosis. Studies on neurodegenerative diseases have indicated that microglia exhibit a stronger response to systemic inflammatory attacks, referred to as ‘microglial priming’ [11, 38]. Similar phenomena may be experienced by elderly patients undergoing surgery.

PA has been shown to improve age-related cognitive impairment. Lack of PA ranks the fourth leading cause of global mortality [39]. Active PA can help alleviate or delay age-related cognitive decline, dementia, Alzheimer’s disease, and other conditions [16, 40]. Even low levels of PA have been reported to reduce the risk of insomnia, sleep disorders, and cognitive decline [41, 42]. In a meta-analysis of 15 international cohorts, it was found that an increase in daily steps was associated with a gradual decrease in the risk of all-cause mortality [43]. Our findings also suggest that for elderly surgical patients, even moderate levels of preoperative PA may have protective effects on early postoperative cognitive function.

PA has been shown to have beneficial effects on cognitive function, which may be associated with a reduction in systemic inflammation levels. In older adults, exercise has been demonstrated to decrease levels of inflammatory biomarkers, thereby reducing the risk of age-related diseases [44]. A systematic review and meta-analysis of 11 randomized controlled trials found that aerobic exercise in middle-aged and older adults was associated with lower levels of inflammatory markers [45]. However, some studies have reported that exercise reduces CRP and IL-6 levels in older adults, but no statistical significance was found for TNF-α [46]. Our study results indicate that the correlation between PA and dNCR is primarily mediated by IL-6, while the statistical significance of TNF-α and IL-1β is not pronounced, possibly due to the limited size of our sample.

What type of PA should be recommended for elderly patients during the perioperative period? The guidelines from the American College of Sports Medicine [47] and the World Health Organization [48] suggest that in the elderly population, PA refers to any activity that results in energy expenditure. This typically includes activities such as housework, shopping, and gardening, rather than organized exercise programs. PA holds significant importance for health-related outcomes in elderly patients, such as falls, fall-related injuries, physical function, frailty, and osteoporosis. In our study, the results of the PASE score categorized by hierarchical clustering indicate that elderly patients, even if they actively engage in daily household chores, such as active engagement in gardening and caring for others as shown in Supplementary Fig. 1, can still exert a protective effect on postoperative cognitive function. Therefore, we should encourage elderly patients undergoing elective surgery to actively participate in daily household chores and engage in physical activities within their capabilities. Both can promote postoperative recovery and reduce the occurrence of complications.

This is a prospective nested case-control study, carefully designed to control for potential confounders from the outset, thereby strengthening the inference of a causal relationship between PA and dNCR. The significance of this study lies in its novel perspective and approach for the prediction, assessment, and prevention of dNCR in elderly patients through preoperative PA. Moreover, the PA we explored is not high-intensity specialized training in sports, which is difficult for elderly patients to achieve, but rather the daily basic household chores that elderly patients are capable of performing. Additionally, through mediation analysis, a preliminary exploration of its mechanism of action was conducted, revealing that PA may exert a protective effect by reducing systemic levels of pro-inflammatory cytokines.

However, the study has several limitations. First, it is an exploratory, single-center study with a limited sample size. Despite efforts to adjust for known confounders at baseline, there may still be residual factors that were not controlled for. Second, the study primarily focuses on elderly patients undergoing total knee arthroplasty under general anesthesia with nerve block to observe the effect of PA on dNCR. It is important to note that this model may not be generalizable to a broader elderly population or patients undergoing different types of surgery. The primary objective of this study is to preliminarily explore the association between moderate-to-low levels of physical activity and delirium in the postoperative period. Third, the assessment of PA is based on the PASE scale, which relies largely on patient self-report, potentially introducing measurement bias. It is expected that future large-scale, multicenter studies will use more objective measures to assess PA. However, the simplicity and convenience of this scale make it advantageous for implementation in clinical practice, providing immediate information. In addition, this study primarily targets three commonly studied pro-inflammatory cytokines as markers of inflammation, with serum samples measured only preoperatively and on the first postoperative day. Future research could include other serologic indicators related to PA to explore potential mechanisms.

## Conclusions

PA exerts a protective effect against dNCR, possibly by reducing serum pro-inflammatory cytokines, particularly IL-6. In elderly patients, engaging in active PA before surgery, even if it is daily household chores, may have a protective effect on postoperative cognitive function.

## Electronic supplementary material

Below is the link to the electronic supplementary material.

Supplementary Material 1

Supplementary Material 2

Supplementary Material 3

## Data Availability

No datasets were generated or analysed during the current study.

## References

[1] Holecki T, Rogalska A, Sobczyk K et al (2020) Global Elderly migrations and their impact on Health Care systems. Front Public Health 8:386. 10.3389/fpubh.2020.00386 32984233 10.3389/fpubh.2020.00386 PMC7477113

[2] Weiser TG, Haynes AB, Molina G et al (2016) Size and distribution of the global volume of surgery in 2012. Bull World Health Organ 94:201–209. 10.2471/BLT.15.159293. F 26966331 10.2471/BLT.15.159293 PMC4773932

[3] Evered L, Silbert B, Knopman DS et al (2018) Recommendations for the nomenclature of cognitive change associated with anaesthesia and surgery—2018. Br J Anaesth 121:1005–1012. 10.1016/j.bja.2017.11.087 30336844 10.1016/j.bja.2017.11.087 PMC7069032

[4] Moller J, Cluitmans P, Rasmussen L et al (1998) Long-term postoperative cognitive dysfunction in the elderly: ISPOCD1 study. Lancet 351:857–861. 10.1016/S0140-6736(97)07382-0 9525362 10.1016/S0140-6736(97)07382-0

[5] Monk TG, Weldon BC, Garvan CW et al (2008) Predictors of cognitive dysfunction after major noncardiac surgery. Anesthesiology 108:18–30. 10.1097/01.anes.0000296071.19434.1e 18156878 10.1097/01.anes.0000296071.19434.1e

[6] Hapca S, Guthrie B, Cvoro V et al (2018) Mortality in people with dementia, delirium, and unspecified cognitive impairment in the general hospital: prospective cohort study of 6,724 patients with 2 years follow-up. Clin Epidemiol 10:1743–1753. 10.2147/CLEP.S174807 30538578 10.2147/CLEP.S174807 PMC6257080

[7] Kainz E, Juilfs N, Harler U et al (2023) The impact of cognitive reserve on delayed neurocognitive recovery after major non-cardiac surgery: an exploratory substudy. Front Aging Neurosci 15:1267998. 10.3389/fnagi.2023.1267998 38076537 10.3389/fnagi.2023.1267998 PMC10701404

[8] Li Z, Zhu Y, Kang Y et al (2022) Neuroinflammation as the underlying mechanism of postoperative cognitive dysfunction and therapeutic strategies. Front Cell Neurosci 16(843069). https://doi.org/10/gqxw2s 10.3389/fncel.2022.843069 PMC8995749 35418837

[9] Lin S-Y, Wang Y-Y, Chang C-Y et al (2021) TNF-α Receptor Inhibitor Alleviates Metabolic and Inflammatory Changes in a Rat Model of Ischemic Stroke. Antioxidants (Basel) 10:851. https://doi.org/10/gn3vs5 10.3390/antiox10060851 PMC8228519 34073455

[10] Han P, Wei L, Duan Z et al (2018) Contribution of IL-1β, 6 and TNF-α to the form of post-traumatic osteoarthritis induced by idealized anterior cruciate ligament reconstruction in a porcine model. Int Immunopharmacol 65:212–220. 10.1016/j.intimp.2018.10.007 30317108 10.1016/j.intimp.2018.10.007

[11] Saxena S, Kruys V, Vamecq J, Maze M (2021) The role of Microglia in Perioperative Neuroinflammation and Neurocognitive disorders. Front Aging Neurosci 13:671499. 10.3389/fnagi.2021.671499 34122048 10.3389/fnagi.2021.671499 PMC8193130

[12] Yang T, Velagapudi R, Terrando N (2020) Neuroinflammation after surgery: from mechanisms to therapeutic targets. Nat Immunol 21:1319–1326. 10.1038/s41590-020-00812-1 33077953 10.1038/s41590-020-00812-1 PMC7704062

[13] Wan Y, Xu J, Ma D et al (2007) Postoperative impairment of cognitive function in rats: a possible role for cytokine-mediated inflammation in the Hippocampus. Anesthesiology 106:436–443. 10.1097/00000542-200703000-00007 17325501 10.1097/00000542-200703000-00007

[14] Radak Z, Torma F, Berkes I et al (2019) Exercise effects on physiological function during aging. Free Radic Biol Med 132:33–41. 10.1016/j.freeradbiomed.2018.10.444 30389495 10.1016/j.freeradbiomed.2018.10.444

[15] Amuthavalli Thiyagarajan J, Mikton C, Harwood RH et al (2022) The UN Decade of healthy ageing: strengthening measurement for monitoring health and wellbeing of older people. Age Ageing 51:afac147. 10.1093/ageing/afac147 35776669 10.1093/ageing/afac147 PMC9249069

[16] Hallal PC, Andersen LB, Bull FC et al (2012) Global physical activity levels: surveillance progress, pitfalls, and prospects. Lancet 380:247–257. 10.1016/S0140-6736(12)60646-1 22818937 10.1016/S0140-6736(12)60646-1

[17] Sewell KR, Erickson KI, Rainey-Smith SR et al (2021) Relationships between physical activity, sleep and cognitive function: a narrative review. Neurosci Biobehavioral Reviews 130:369–378. 10.1016/j.neubiorev.2021.09.003 10.1016/j.neubiorev.2021.09.003 34506842

[18] Liu Y, Chu JMT, Ran Y et al (2022) Prehabilitative resistance exercise reduces neuroinflammation and improves mitochondrial health in aged mice with perioperative neurocognitive disorders. J Neuroinflammation 19(150). https://doi.org/10/gsj7gn 10.1186/s12974-022-02483-1 PMC9199135 35705955

[19] Son W-H, Park H-T, Jeon BH, Ha M-S (2023) Moderate intensity walking exercises reduce the body mass index and vascular inflammatory factors in postmenopausal women with obesity: a randomized controlled trial. Sci Rep 13:20172. 10.1038/s41598-023-47403-2 37978254 10.1038/s41598-023-47403-2 PMC10656478

[20] Sabouri M, Kordi M, Shabkhiz F, et al et al (2020) Moderate treadmill exercise improves spatial learning and memory deficits possibly via changing PDE-5, IL-1 β and pCREB expression. Exp Gerontol 139:111056. https://doi.org/10/gq6rmm 32791334 10.1016/j.exger.2020.111056

[21] Vaughan K, Miller WC (2013) Validity and reliability of the Chinese translation of the physical activity scale for the Elderly (PASE). Disabil Rehabil 35:191–197. 10.3109/09638288.2012.690498 22671717 10.3109/09638288.2012.690498 PMC3540101

[22] Washburn RA, Smith KW, Jette AM, Janney CA (1993) The physical activity scale for the Elderly (PASE): development and evaluation. J Clin Epidemiol 46:153–162. 10.1016/0895-4356(93)90053-4 8437031 10.1016/0895-4356(93)90053-4

[23] Rasmussen LS, Larsen K, Houx P et al (2001) The assessment of postoperative cognitive function. Acta Anaesthesiologica Scandinavica 45:275–289. https://doi.org/10/brxdqg 10.1034/j.1399-6576.2001.045003275.x 11207462

[24] Johnson T, Monk T, Rasmussen LS et al (2002) Postoperative cognitive dysfunction in Middle-aged patients. Anesthesiology 96:1351–1357. 10.1097/00000542-200206000-00014 12170047 10.1097/00000542-200206000-00014

[25] Brand N, Jolles J (1985) Learning and Retrieval Rate of words presented auditorily and visually. J Gen Psychol 112:201–210. 10.1080/00221309.1985.9711004 4056765 10.1080/00221309.1985.9711004

[26] Guo Y (2022) A selective review of the ability for variants of the trail making test to assess cognitive impairment. Appl Neuropsychology: Adult 29:1634–1645. 10.1080/23279095.2021.1887870 10.1080/23279095.2021.1887870 33625945

[27] Jaeger J (2018) Digit symbol substitution test. J Clin Psychopharmacol 38:513–519. 10.1097/JCP.0000000000000941 30124583 10.1097/JCP.0000000000000941 PMC6291255

[28] Scarpina F, Tagini S (2017) The Stroop Color and Word Test. Front Psychol 8. 10.3389/fpsyg.2017.00557 10.3389/fpsyg.2017.00557 PMC5388755 28446889

[29] McDonagh DL, Phillips-Bute B, Newman MF (2010) Cognitive function after major noncardiac surgery, apolipoprotein E4 genotype, and biomarkers of Brain Injury. PERIOPERATIVE Med 112 10.1097/ALN.0b013e3181d31fd7 PMC2933423 20216394

[30] Hughes CG, Boncyk CS, Culley DJ et al (2020) American Society for Enhanced Recovery and Perioperative Quality Initiative Joint Consensus Statement on Postoperative Delirium Prevention. Anesth Analg 130:1572–1590. 10.1213/ANE.0000000000004641 32022748 10.1213/ANE.0000000000004641 PMC7379173

[31] Peden CJ, Miller TR, Deiner SG et al (2021) Improving perioperative brain health: an expert consensus review of key actions for the perioperative care team. Br J Anaesth 126:423–432. 10.1016/j.bja.2020.10.037 33413977 10.1016/j.bja.2020.10.037

[32] Digitale JC, Martin JN, Glymour MM (2022) Tutorial on Directed Acyclic Graphs. J Clin Epidemiol 142:264–267. 10.1016/j.jclinepi.2021.08.001 34371103 10.1016/j.jclinepi.2021.08.001 PMC8821727

[33] Lee SS, Lo Y, Verghese J (2019) Physical activity and risk of postoperative delirium. J Am Geriatr Soc 67:2260–2266. 10.1111/jgs.16083 31368511 10.1111/jgs.16083 PMC6861610

[34] Cheng C, Wan H, Cong P et al (2022) Targeting neuroinflammation as a preventive and therapeutic approach for perioperative neurocognitive disorders. J Neuroinflammation 19:297. 10.1186/s12974-022-02656-y 36503642 10.1186/s12974-022-02656-y PMC9743533

[35] Zhou Q, Yu L, Yin C et al (2022) Effect of transcutaneous auricular vagus nerve stimulation on delayed neurocognitive recovery in elderly patients. Aging Clin Exp Res 34:2421–2429. 10.1007/s40520-022-02177-x 35809206 10.1007/s40520-022-02177-x

[36] Chen A-Q, Fang Z, Chen X-L et al (2019) Microglia-derived TNF-α mediates endothelial necroptosis aggravating blood brain–barrier disruption after ischemic stroke. Cell Death Dis 10:487. 10.1038/s41419-019-1716-9 31221990 10.1038/s41419-019-1716-9 PMC6586814

[37] Yang S, Gu C, Mandeville ET et al (2017) Anesthesia and surgery impair blood–brain barrier and cognitive function in mice. Front Immunol 8:902. 10.3389/fimmu.2017.00902 28848542 10.3389/fimmu.2017.00902 PMC5552714

[38] Luo G, Wang X, Cui Y et al (2021) Metabolic reprogramming mediates hippocampal microglial M1 polarization in response to surgical trauma causing perioperative neurocognitive disorders. J Neuroinflammation 18(267). https://doi.org/10/gp65st 10.1186/s12974-021-02318-5 PMC8590219 34774071

[39] Kohl HW, Craig CL, Lambert EV et al (2012) The pandemic of physical inactivity: global action for public health. Lancet 380:294–305. 10.1016/S0140-6736(12)60898-8 22818941 10.1016/S0140-6736(12)60898-8

[40] Cacciatore F, Amarelli C, Ferrara N et al (2019) Protective effect of physical activity on mortality in older adults with advanced chronic heart failure: A prospective observational study. Eur J Prev Cardiolog 26:481–488. https://doi.org/10/grtj4j 10.1177/2047487318790822 30066588

[41] Sanders LMJ, Hortobágyi T, Karssemeijer EGA et al (2020) Effects of low- and high-intensity physical exercise on physical and cognitive function in older persons with dementia: a randomized controlled trial. Alzheimers Res Ther 12:28. 10.1186/s13195-020-00597-3 32192537 10.1186/s13195-020-00597-3 PMC7082953

[42] Galle SA, Deijen JB, Milders MV et al (2023) The effects of a moderate physical activity intervention on physical fitness and cognition in healthy elderly with low levels of physical activity: a randomized controlled trial. Alzheimers Res Ther 15:12. 10.1186/s13195-022-01123-3 36631905 10.1186/s13195-022-01123-3 PMC9832427

[43] Paluch AE, Bajpai S, Bassett DR et al (2022) Daily steps and all-cause mortality: a meta-analysis of 15 international cohorts. Lancet Public Health 7:e219–e228. 10.1016/S2468-2667(21)00302-9 35247352 10.1016/S2468-2667(21)00302-9 PMC9289978

[44] Rose GL, Skinner TL, Mielke GI, Schaumberg MA (2021) The effect of exercise intensity on chronic inflammation: a systematic review and meta-analysis. J Sci Med Sport 24:345–351. 10.1016/j.jsams.2020.10.004 33153926 10.1016/j.jsams.2020.10.004

[45] Gaitán JM, Moon HY, Stremlau M et al (2021) Effects of Aerobic Exercise training on systemic biomarkers and cognition in late middle-aged adults at risk for Alzheimer’s Disease. Front Endocrinol 12(660181). https://doi.org/10/gnjkq4 10.3389/fendo.2021.660181 PMC8173166 34093436

[46] Monteiro-Junior RS, De Tarso Maciel-Pinheiro P, Da Matta Mello Portugal E et al (2018) Effect of Exercise on Inflammatory Profile of older persons: systematic review and Meta-analyses. J Phys Activity Health 15:64–71. 10.1123/jpah.2016-0735 10.1123/jpah.2016-0735 28771081

[47] Chodzko-Zajko WJ, Proctor DN, Fiatarone Singh MA et al (2009) Exercise and physical activity for older adults. Med Sci Sports Exerc 41:1510. 10.1249/MSS.0b013e3181a0c95c 19516148 10.1249/MSS.0b013e3181a0c95c

[48] Bull FC, Al-Ansari SS, Biddle S et al (2020) World Health Organization 2020 guidelines on physical activity and sedentary behaviour. Br J Sports Med 54:1451–1462. 10.1136/bjsports-2020-102955 33239350 10.1136/bjsports-2020-102955 PMC7719906

