# Supplementary material for: The association between physical activity and delayed neurocognitive recovery in elderly patients: a mediation analysis of pro-inflammatory cytokines
Source: Aging Clin Exp Res. 2024 Sep 11;36(1):192. doi: 10.1007/s40520-024-02846-z (PMC11390811; doi:10.1007/s40520-024-02846-z)

**Supplementary Fig. 1** The second-order cluster diagram of PASE scores.

(a) The characteristics and quality of the second-order clustering model; (b) The features of the two types of physical activity clusters after clustering; (c) The ranking of the proportion of each activity type from highest to lowest.


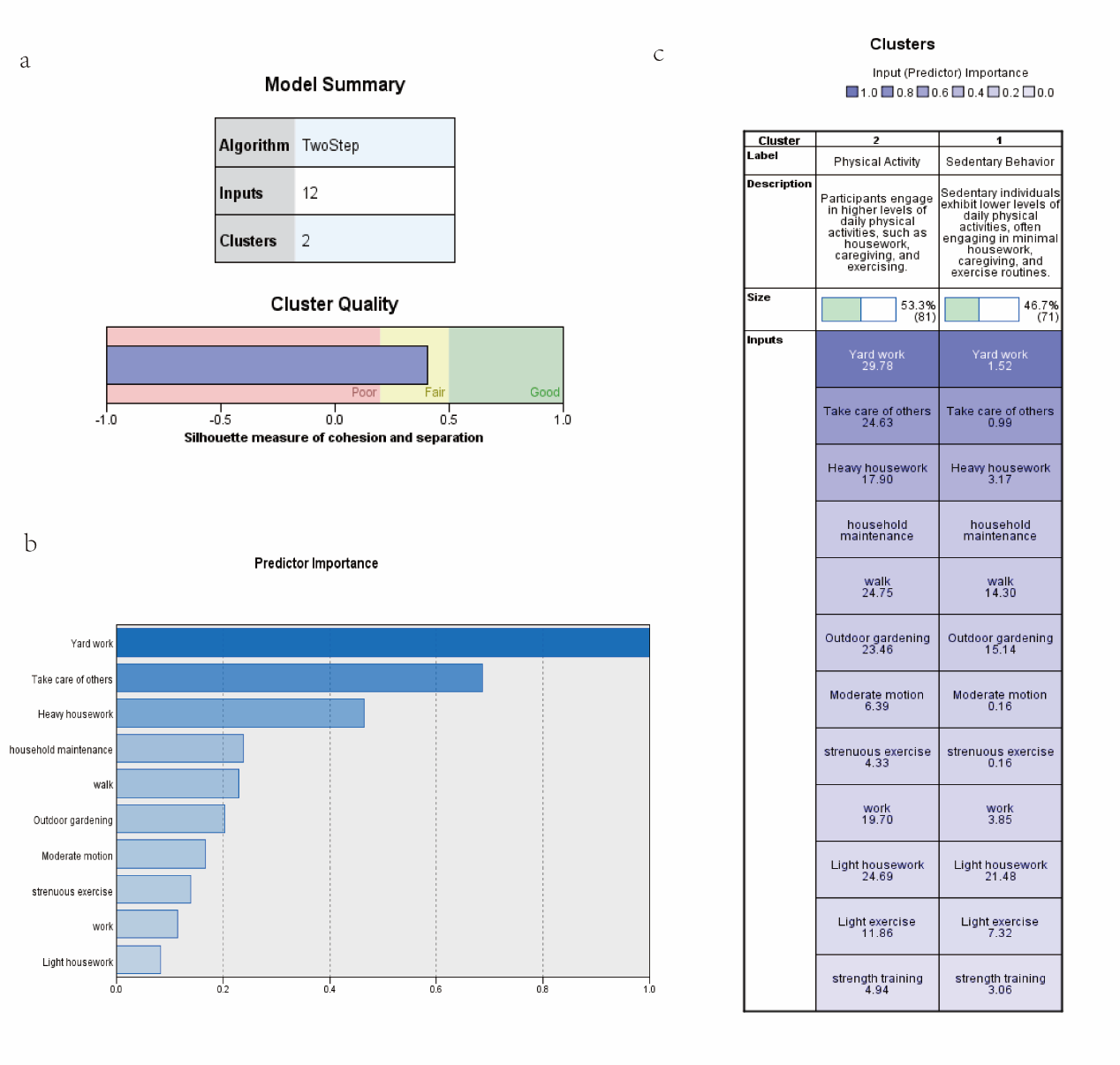

Supplement: Supplementary file 1 — Supplementary Material 1 [file 40520_2024_2846_MOESM1_ESM.docx]
