# Supplementary material for: The association between physical activity and delayed neurocognitive recovery in elderly patients: a mediation analysis of pro-inflammatory cytokines
Source: Aging Clin Exp Res. 2024 Sep 11;36(1):192. doi: 10.1007/s40520-024-02846-z (PMC11390811; doi:10.1007/s40520-024-02846-z)

**Supplementary Fig. 2** Analysis of the receiver operating characteristic for the predictive value of dNCR.


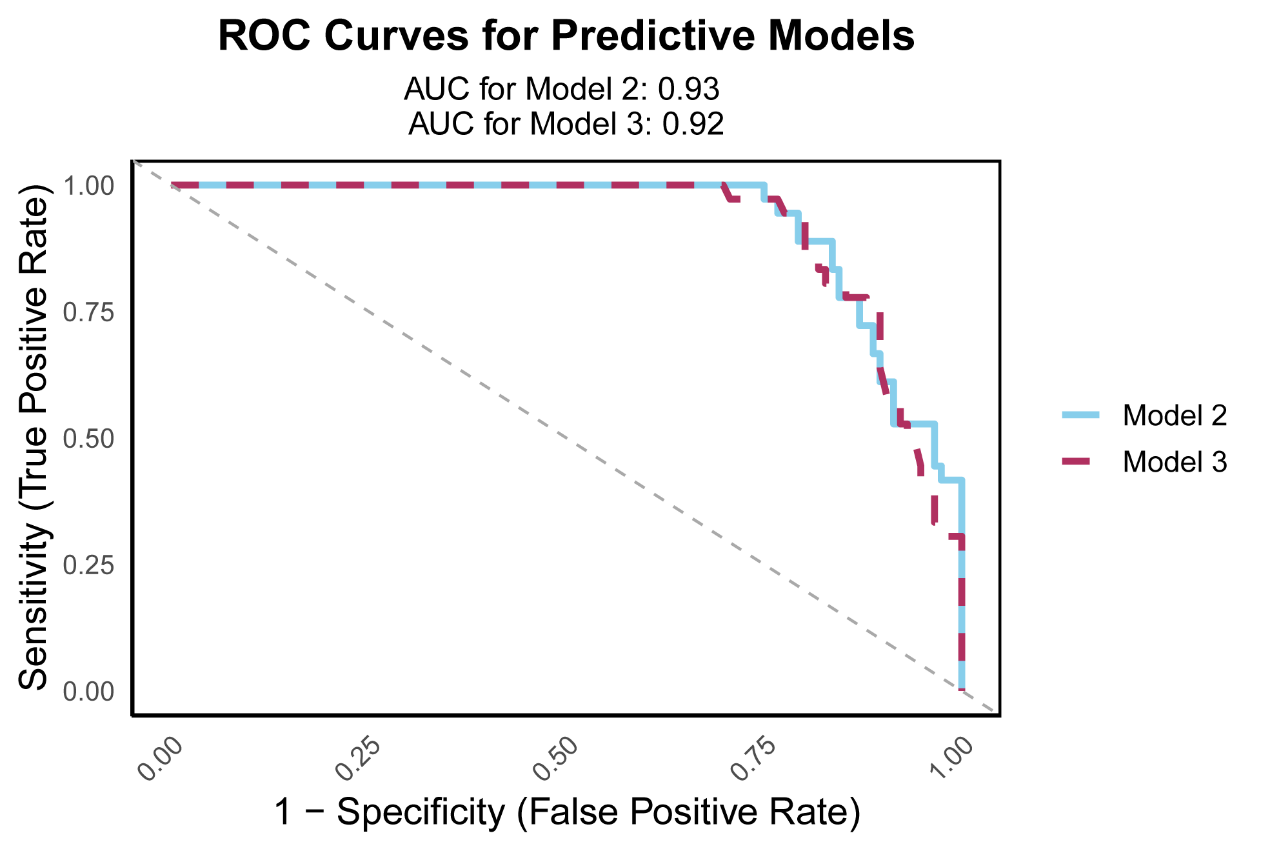

Supplement: Supplementary file 2 — Supplementary Material 2 [file 40520_2024_2846_MOESM2_ESM.docx]
