# Supplementary material for: The association between physical activity and delayed neurocognitive recovery in elderly patients: a mediation analysis of pro-inflammatory cytokines
Source: Aging Clin Exp Res. 2024 Sep 11;36(1):192. doi: 10.1007/s40520-024-02846-z (PMC11390811; doi:10.1007/s40520-024-02846-z)
Supplement: Supplementary file 3 — Supplementary Material 3 [file 40520_2024_2846_MOESM3_ESM.docx]

**Supplementary Table 1: S**ensitivity analysis: Multivariate logistic regression model showing associations between PA and dNCR

|  | Model 4 | | Model 5 | | Model 6 | |
| --- | --- | --- | --- | --- | --- | --- |
|  | Adjusted OR (95% CI) | *P* | Adjusted OR (95% CI) | *P* | Adjusted OR (95% CI) | *P* |
| Age | 1.228 (1.071, 1.409) | **0.003** | 1.224 (1.065, 1.407) | **0.005** | 1.233 (1.039, 1.464) | **0.017** |
| CCI | 2.353 (1.360, 4.071) | **0.002** | 2.574 (1.442, 4.595) | **0.001** | 2.407 (1.324, 4.377) | **0.004** |
| CES-D 10 | 2.358 (1.535, 3.622) | **<0.001** | 2.529 (1.621, 3.945) | **<0.001** | 3.123 (1.689, 5.775) | **<0.001** |
| ASA | 4.542 (0.921, 22.403) | 0.063 | 3.990 (0.789, 20.171) | 0.094 | 5.116 (0.820, 31.927) | 0.081 |
| PASE score median | 0.229 (0.069, 0.759) | **0.016** | - | **-** | - | - |
| PASE score: moderate | - | **-** | 0.260 (0.077, 0.879) | **0.030** | - | - |
| PASE score: high | - | **-** | 0.110 (0.021, 0.569) | **0.008** | - | - |
| PASE score (age≥65) | - | **-** | - | - | 0.985 (0.975, 0.995) | **0.004** |

**Abbreviations** PA: physical activity; dNCR, dNCR, delayed neurocognitive recovery; CCI: Charlson Comorbidity Index; CES-D 10: the 10-item Center for Epidemiologic Studies Depression Scale; ASA: American Society of Anesthesiologists; PASE: physical activity scale for the elderly; CI: confidence interval; OR: odds ratio.

**Model 4:** Multivariate logistic regression analysis was conducted with the PASE score categorized into two groups based on the median, adjusting for confounding factors such as age, CCI, CES-D 10, and ASA. Boldface values indicate *P* < 0.05.

**Model 5:** Multivariate logistic regression analysis was conducted with the PASE score divided into three groups based on tertiles, adjusting for age, CCI, CES-D 10, and ASA.

**Model 6**: Multivariate logistic regression analysis was conducted excluding patients younger than 65 years old, using the PASE score as a continuous variable, and adjusting for age, CCI, CES-D 10, and ASA.
